# Correspondence

**Published:** 1875-06

**Authors:** 


### Correspondence.

Through the kindness of Prof. White, we are placed in possession of the following letter, which gives the history of three interesting cases of inversio uteri. The means proposed for the cure of Case I. were intelligently conceived, and as far as the timid husband would permit, well carried out; we only regret that the efforts could not have been continued to a successful termination, which, we have no doubt, would have been the case had not the husband interfered. Case II. is remarkable from the long continuation of the invertion; we have no recollection of any case having been reported of that duration; the nearest approach to it being a case of twenty-two years duration, reduced by Prof. White,* and one of twenty-six years, referred to by Dr. Evory Kennedy, in the transactions of the Dublin Obstetrical Society,† which had been allowed to go unreduced. It is to be regretted, that the advice of the ignorant quack, who was the cause of the inversion in Case III., induced the patient to forego any attempt at reduction. We have been very much interested in the report, and have no doubt but that our readers will also. [Ed.]

Prof. James P. White, M. D., Buffalo, N. Y.

*Dear Sir:*—Since exciting your curiosity in regard to my observations of cases of *inversio uteri*, I have been sick of pneumonia, and am not now able to leave the house, which will account for the delay of this communication. My cases are now only interesting to the pathologist, and historian; and illustrative of the many vexations incident to our profession.

I shall relate them in the chronological order in which they came to my knowledge, to-wit:

Case I. In April, 1861, I was called to see, in this City, Mrs. C., æt. 31 years, German; wife of a Gardener, fairly intelligent; had borne one child, in the State of Ohio, a daughter, about 11 years previously. She informed me that her *Accoucheur* gave her great pain in removing the "after-birth," and, that she had never since been free from an unnatural body in the vagina; nor from profuse vaginal fluxes.

At each menstrual epoch, the hæmorrhage was profuse and frequently alarming. Herself and husband, both, had correct ideas of the condition, but only asked and expected a local styptic.

She was anæmic—"waxy"—and slightly anasarcous, appetite good, and digestion without pain. Her strength was sufficient to attend to part of her household duties. This had not been true until within the last few years.

Examination in every method thought of, or recorded, was perfectly practicable; presented every sign and symptom, of inverted uterus; and I will not consume time in reporting details.

* Buffalo Medical and Surgical Journal, August, 1872. Case No. X.
† Obstetrical Journal Great Britain and Ireland, vol. I., No. 5, page 341.

Of course strong encouragement was given to the sufferer and her husband. Your case, as reported in "*Buffalo Med. Journal,*" and the case of Dr. Tyler Smith were cited; and a day set for the attempt.

The husband objected, positively, to the presence of more than one Professional and two female attendants. On the appointed day, assisted by the late Dr. Henry B. Moore, formerly of Manlius, N. Y., whose experience and professional ability left nothing to be desired, we made an attempt at reduction. Anæsthæsia was readily induced by inhalation of chloroform and manipulation commenced; when the busband became alarmed, without any reason that we, or the non-professional attendants could discover, and we were compelled to desist; having already made a perceptible indentation in the fundus with my thumb.

Recovery from anæsthæsia was prompt and complete, slight hæmorrhage had been started, but immediately ceased; and, on the succeeding day our patient complained of no result from the effort. The husband seemed ashamed of his needless alarm, and was ready to appoint another day for the operation. Upon that day with the same professional aid; armed with both Ether Sulph. and Chloroform; and with a firm copper rod about fifteen inches long, terminating at one end in a ball of about one inch diameter, and at the other in a hollow tube of about one half inch diameter, which was firmly plugged with wool—sponge projecting in a cone beyond the rim of the cylinder, we commenced under favorable auspices to administer ether, hoping that the husband would have less fears of its effect, but, in this we were mistaken, for he was sooner scared than before, and peremptorily ordered us "stop; you are killing her." My disgust was supreme, and I would never have tried again, unless the husband would *leave*—and *stay* out of the house; but my friend Dr. Moore visited the family several times; and by arguments—reading to them your report, etc.; and ridiculing the husband's timidity, in which he was joined by the patient, brought them to the resolution to have a *thorough* trial made, and of course I yielded to his judgment inspired by confidence in the sufficiency of art.

Waiting for expiration of a menstrual period we assembled armed as before, and with some *etceteras.* May 7th, 1861. Everything went smoothly for about 30 minutes, when the husband's courage again collapsed. Dr. Moore, with one hand upon the abdomen had taken hold of the repositor whilst my hand was resting, preparatory to as vigorous action as the yielding sensations led us to expect would soon be warrantable.

The women, full of confidence, tried in vain to encourage, and to shame him; and all of us appealed to his sympathy for his long-suffering wife; but only temporarily succeeded, and at the end of fifty-five minutes from the introduction of the vaginal hand, the howlings, threats, and frenzy of the coward were irresistible. The fundus was well depressed, the patulous *os* could be felt by Dr. M. through the abdominal wall, and success seemed too near for us to be foiled.

No untoward symptom arose, and the patient expressed deep regret at her husband's "faint heart." In April, 1862, I went into Military service; and on my return in April, 1865, I learned that she had died in the summer of 1864, having lived over fourteen years with the inversio uteri.

Case II. In the winter of 1863–4, while quartered with my brigade near Culpepper C. H., Va., the superintendent of poor for that county invited another surgeon and myself to see some interesting cases in his institution.

One of them was a white woman of feeble intellect, but by no means an idiot, about fifty-five years of age, four and a half feet in height, square built, but now slightly "Grecian," and waddling in gait, leucophlegmatic, but capable of doing considerable light work, whose uterus had been inverted for over thirty years. The accident occurred, she said, "right away after the baby was born." The superintendent had known her for several years, and stated that previous to the menopause she had been extremely feeble and anæmic; having constant fluxes, either muco-purulent or sanguineous; that the uterus was sometimes very much larger than at others; but had always protruded more or less external to the vulva.

We found the uterus completely inverted, and pendent external to the nymphæ, but with its *os* and the vaginal reflex grasped by the labia majora. Its everted surface was perfectly dry, resembling the cuticle on the back of the hand of old women, or somewhat like cicatricial tissue. Since about her forty-eighth year, the uterus had not changed very much from its present size, to wit; length, two and three-quarter inches; lateral diameter at the cornui, one and three-quarter inches; lateral diameter at the cervix, one and one-quarter inches; anterior-posterior at the fundus, one and one-quarter inches; anterior-posterior at the cervix, three-quarter inch.

We could pull the organ downwards, so that the point of an index finger could carry the vaginal wall, either before or behind, into the upward looking os; and could feel the ovaries on either side protruding above.

Micturitition and defecation were normally performed. She said, "the itching and straining used to be dreffful; but I doesn't mind it now."

Case III. August 6th, 1869, was called to see Mrs. V., æt. twenty-six years—American—wife of a "well-to-do" farmer.

She was believed to have some malignant disease of the genital organs, from which she had suffered since the birth of a child, about two years previous.

Herself and husband declared, that her attendant, (an irregular practitioner,) had used great violence in removing the secundines; and, that she had suffered almost constant hæmorrhage since. A great variety of irregular and domestic styptic prescriptions had been used, with but little apparent effect.

She was extremely exsanguined, and somewhat anasarcous. Pulse 142—respiration not rapid—digestion impaired, as usual in anæmia.

There was no difficulty in passing a very large cylindrical speculum; nor in introducing two fingers high into the rectum. The one exposed the intra-uterine surface, now external, the distinct form of the fundus, cornui, and fallopian orifices. The other, by tilting the body of the womb toward the bladder with my left hand, enabled me to feel the depression of the *os* through the intestinal wall.

Such was the extreme relaxation of the sphincters, that digital exploration was unobstructed and nearly painless. I explained, and exhibited the condition to the husband, and to two or three female friends, dressed the everted membrane with a weak solution of *sulph. zinci,* prescribed energetic tonics, and left with a promise that on my visit, two days after, a decision should be made as to time, etc., of an operation.

On account of illness, I could not keep my appointment, but learned that hæmorrhage and most of the leucorrhœa had been stopped by the applications, (or by exhaustion,) and that the patient felt very much better; also, that I was to await a further call. The impertinent quack who had done the mischief, still had the confidence of the woman's relatives; and, had assured them that such an accident as I had represented "could not occur," that any interference with her condition in her weak state, would be suddenly fatal, etc., etc.

The husband had good sense in all things, but the "ruling of his own house"; and the patient had surrendered to despondency, had arranged her affairs for death, and patiently awaited for its relief.

August 29th, twenty-three days after my first visit, I was called again. Found the patient sinking, but still urged an effort to rescue her from certain death, to no avail. She died four days after with asthænia.

Hoping that these pages may prove of some interest to one who has done so much for this unfortunate class of sufferers; and, vouching for the correctness of every essential statement which may tend to make up the history and pathology of the accident, I place at them your service with the highest sentiments of regard.

J. H. BEECH, M. D.

January 27th, 1875. Coldwater, Mich.

